# The intergenerational association of preterm birth: A systematic review and meta‐analysis

**DOI:** 10.1111/1471-0528.17924

**Published:** 2024-08-07

**Authors:** Abdulbasit Seid, Miranda S. Cumpston, Kedir Y. Ahmed, Habtamu Mellie Bizuayehu, Subash Thapa, Teketo Kassaw Tegegne, Abel F. Dadi, Daniel Bogale Odo, Desalegn Markos Shifti, Sewunet Admasu Belachew, Getiye Dejenu Kibret, Daniel Bekele Ketema, Zemenu Yohannes Kassa, Erkihun Amsalu, Meless G. Bore, Tahir Ahmed Hassen

**Affiliations:** ^1^ School of Public Health and Preventive Medicine Monash University Melbourne Victoria Australia; ^2^ Rural Health Research Institute Charles Sturt University Orange New South Wales Australia; ^3^ First Nations Cancer and Wellbeing (FNCW) Research Program, School of Public Health The University of Queensland Herston Queensland Australia; ^4^ Institute for Physical Activity and Nutrition Deakin University Geelong Victoria Australia; ^5^ Menzies School of Health Research Charles Darwin University Casuarina Northern Territory Australia; ^6^ Addis Continental Institute of Public Health Addis Ababa Ethiopia; ^7^ National Centre for Aboriginal and Torres Strait Islander Wellbeing Research, National Centre for Epidemiology And Population Health Australian National University Canberra Australia; ^8^ Child Health Research Centre The University of Queensland South Brisbane Queensland Australia; ^9^ Faculty of Health, School of Public Health University of Technology Sydney Ultimo New South Wales Australia; ^10^ Centre for Health Systems and Safety Research, Australian Institute of Health Innovation, Faculty of Medicine, Health and Human Sciences Macquarie University Sydney New South Wales Australia; ^11^ The George Institute for Global Health University of New South Wales (UNSW) Sydney New South Wales Australia; ^12^ School of Public Health, College of Medicine and Health Science Debre Markos University Debre Markos Ethiopia; ^13^ College of Medicine and Health Sciences Hawassa University Hawassa Ethiopia; ^14^ School of Nursing and Midwifery University of Technology Sydney Sydney New South Wales Australia; ^15^ Faculty of Medicine and Health, Sydney Medical School University of Sydney Sydney New South Wales Australia; ^16^ St. Paul Hospital Millennium Medical College Addis Ababa Ethiopia; ^17^ School of Nursing, College of Medicine and Health Science Hawassa University Hawassa Ethiopia; ^18^ Center for Women's Health Research, College of Health, Medicine and Wellbeing The University of Newcastle Callaghan New South Wales Australia

**Keywords:** intergenerational association, meta‐analysis, preterm birth, systematic review

## Abstract

**Background:**

Around half of preterm births lack identifiable causes, indicating the need for further investigation to understand preterm birth risk factors. Existing studies on the intergenerational association of preterm birth showed inconsistency in effect size and direction.

**Objective:**

This systematic review and meta‐analysis aimed to review existing studies and provide comprehensive evidence on the intergenerational association of preterm births.

**Search Strategy:**

We searched MEDLINE, Embase and Maternity and Infant Care databases, from the inception of each database to 04 April 2024.

**Selection Criteria:**

Eligibility criteria included studies that reported on women who had given birth and had recorded information about a family history of preterm birth in one or both of the child's biological parents.

**Data Collection and Analysis:**

Data were extracted by two independent reviewers. A random‐effects model was used to compute pooled estimates using odds ratios.

**Main Results:**

Sixteen eligible studies with a total of 2 271 612 mothers were included. The findings indicated a 1.44 (OR = 1.44, 95% CI: 1.34, 1.54) fold increase in odds of giving preterm births among women who were born preterm. Additionally, having a sibling born preterm (OR = 1.53, 95% CI: 1.24, 1.87) and having a partner born preterm (OR = 1.12, 95% CI: 1.01, 1.25) were associated with increased likelihood of giving preterm births among women.

**Conclusion:**

The study revealed that women with a family history of preterm birth face an increased risk of giving preterm births. Screening pregnant women for a family history of preterm birth is essential, with those having a positive family history requiring closer follow‐up.

## 1 INTRODUCTION

Preterm birth, a birth occurring before 37 weeks of gestation, poses a substantial global health challenge, affecting nearly 15 million infants annually and contributing to over a million infant deaths.^1^ Preterm birth accounts for 35% of all neonatal deaths and 18% of deaths among under‐five children.^2^ In addition to the devastating loss of life due to preterm birth, premature infants often experience significant developmental complications, encompassing neurosensory deficits and learning impairments, that can persist throughout their lives.^2^, ^3^

Advancements in research have led to effective treatments that improve preterm outcomes such as administration of antenatal steroids to enhance lung maturity,^4^ tocolytic treatments to delay labor,^5^ and prophylactic antibiotics for premature rupture of membranes.^6^ While these treatments prevent the long‐term repercussions of prematurity, they do not address the primary prevention of premature birth. Currently, as part of primary prevention of preterm birth, interventions, such as progesterone administration, and cervical cerclage are being implemented.^7^ However, these interventions are only effective when targeted at women with identifiable risks of preterm birth.^7^ For instance, most existing guidelines recommend progesterone and/or cerclage for women with specific risk factors, including a history of preterm birth or cervical trauma.^8^ Yet, as only about half of preterm births have identifiable causes, identifying high‐risk groups solely based on limited risk factors remains challenging.^9^

In expanding the understanding of these risk factors, the potential intergenerational association of preterm birth has become a focal point of recent investigations.^10^, ^11^ However, existing epidemiological studies that investigated the intergenerational association of preterm reported conflicting results,^12^, ^13^, ^14^, ^15^ necessitating a systematic review and meta‐analysis. Therefore, this review aimed to pool the existing evidence on the intergenerational association of preterm birth and provide evidence to inform interventions aimed at reducing preterm birth.

## 2 METHODS

### 2.1 Registration

The protocol for this review was registered in the International Prospective Register of Systematic Reviews (CRD42024496813).^16^ The review was conducted and reported in accordance with Preferred Reporting Items for Systematic Reviews and Meta‐Analyses (PRISMA).^17^

### 2.2 Data source and search strategy

Our electronic database search included the OVID Medline, OVID Embase and OVID Maternity and Infant Care databases. The search encompassed from the inception of each database and was conducted on 05 January 2024, with a subsequent update on 04 April 2024. The search strategy was developed by the research team members with extensive experience in conducting effective searching using different medical databases. Search terms such as preterm birth, parents, mothers, fathers, families, generational and intergenerational were used with their related synonyms, and combined with appropriate Boolean operators. The search was not restricted by study setting or year of publication. Details of the search are included in Appendix S1.

### 2.3 Eligibility criteria

The review included published original observational studies reporting on the intergenerational association of preterm birth. The study population consisted of women who had given birth to a child and whose information about their own or their partner's family history of preterm birth had been documented. A family history of preterm birth (i.e. exposure) was characterised by any reported occurrences of preterm birth among parents or other close relatives of either of the child's biological parents. The comparators were biological parents with no family history of preterm birth. Preterm birth among the women under investigation (i.e. outcome) was defined as a baby born before 37 weeks of gestation.^2^ All studies reporting on preterm births, regardless of whether they were spontaneous or iatrogenic, were included. Studies exclusively focusing on multiple pregnancies and those that reported on family history of preterm birth without specifying the family member's relationship to the preterm child were excluded.

### 2.4 Selection of articles

All identified articles were exported to Endnote Version 20 and non‐duplicate articles were exported to Covidence for screening. Each article was screened by two independent reviewers (AS and TH) from the research team by titles and abstracts to remove irrelevant studies. Then, full texts of the remaining studies were retrieved and again screened for eligibility by two reviewers (AS and TH). Any disagreements were resolved through consultation.

### 2.5 Data extraction and quality assessment

Data were extracted by two independent reviewers (AS and TH) using a prepared and pre‐piloted data extraction tool which was specifically designed to capture all relevant information. Quality assessment of the included studies was independently conducted by two reviewers (AS and TH) using the Joanna Briggs Institute (JBI) Critical Appraisal Checklists designed for cohort and case–control studies.^18^ The risk of bias for each included study was classified as low (when a study received ‘Yes’ responses on 70% of the items), moderate (when received ‘Yes’ responses on 50–69% of the items) and high (when received ‘Yes’ responses on less than 50% of the items), as used in previous studies.^19^, ^20^ Any disagreement between the two independent reviewers during data extraction or quality appraisal was resolved through discussion.

### 2.6 Data synthesis

A summary table depicting the characteristics of each included study was created to describe the individual studies. To investigate the association between family history of preterm birth and preterm birth among mothers, pooled odds ratios (OR) with 95% confidence intervals (CI) were computed using the ORs (preferably adjusted ORs) which were obtained from the original studies or calculated using reported raw data, where ORs were not readily available. Stata version 16 software was used, employing the ‘*metan*’ command to calculate the pooled effect size. Random‐effects model using the DerSimonian–Laird (DL) estimator was chosen to account for significant heterogeneity among studies. The DL method was relatively simple to compute and is standard estimator programmed into many meta‐analysis software packages including Stata. The level of heterogeneity was expressed using the *I* ^2^ statistic, and it was considered significant when the chi‐square test yielded a *p*‐value of <0.1 as recommended by Cochrane.^21^ A subgroup analysis based on study design and adjustment of confounders was conducted to explore the heterogeneity. Furthermore, a sensitivity analysis was planned using ‘leave one out’ approach to examine the robustness of the findings.

The risk of publication bias was assessed using funnel plots when the number of included studies was sufficient (ten or more), and Egger's test was used for quantitative evaluation.^22^ Where a single study reported on the family history of preterm birth stratified by other specific population characteristics (e.g. race), without presenting the overall effect, the effect size from each stratified analysis was included in the pooled analysis.^23^ However, when a single study reported on various subtypes of preterm birth (e.g. extreme, very or moderate preterm), without reporting an overall preterm birth outcome, the effect size with the most precise estimate was selected for inclusion in the pooled analysis.

## 3 RESULTS

**FIGURE 1:**
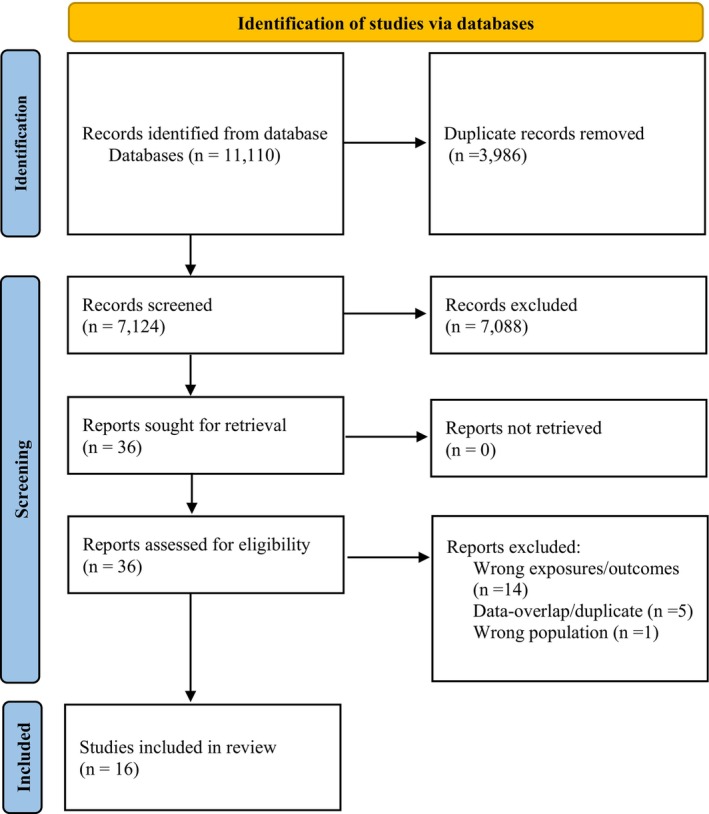
PRISMA flow chart for included studies.

The overall search yielded a total of 11 110 articles. After removing the duplicates, 7124 articles were screened using abstracts and titles, of which 7088 were excluded. The remaining 36 articles underwent further full‐text screening, with final inclusion of 16 studies (Figure 1). The list of excluded studies is available as Table S1.

### 3.1 Characteristics of included studies

Overall, the studies included a total of 2 271 612 mothers. Nearly all the studies, with the exception of one from Iran,^24^ were conducted in higher‐income countries. Six studies originated from the United States,^15^, ^23^, ^25^, ^26^, ^27^, ^28^ two studies each from Norway^29^, ^30^ and Denmark,^31^, ^32^ while the remaining studies were from United Kingdom,^12^ Canada,^33^ Sweden,^13^ Italy^34^ and Israel.^14^ The majority of included studies were published after 2010, with only six exceptions.^28^, ^29^, ^30^, ^31^, ^32^, ^35^ Apart from two case–control studies,^24^, ^34^ all included studies were population‐based retrospective cohort studies (Table S2).

### 3.2 Quality of included studies

Overall, out of the 16 included studies, eight were determined to have a low risk of bias,^12^, ^15^, ^23^, ^24^, ^29^, ^30^, ^31^, ^34^ seven were considered to have a moderate risk of bias,^13^, ^14^, ^25^, ^26^, ^27^, ^32^, ^33^ and one was classified as having a high risk of bias.^28^ Among the cohort studies, six were determined to have a low risk of bias,^12^, ^15^, ^23^, ^29^, ^30^, ^31^ seven had a moderate risk of bias,^13^, ^14^, ^25^, ^26^, ^27^, ^32^, ^33^ and one was classified as having a high risk of bias^28^ (Table S3). All case–control studies were found to have a low risk of bias^24^, ^34^ (Table S4).

### 3.3 Intergenerational association of preterm birth

#### 3.3.1 Mother born preterm

**FIGURE 2:**
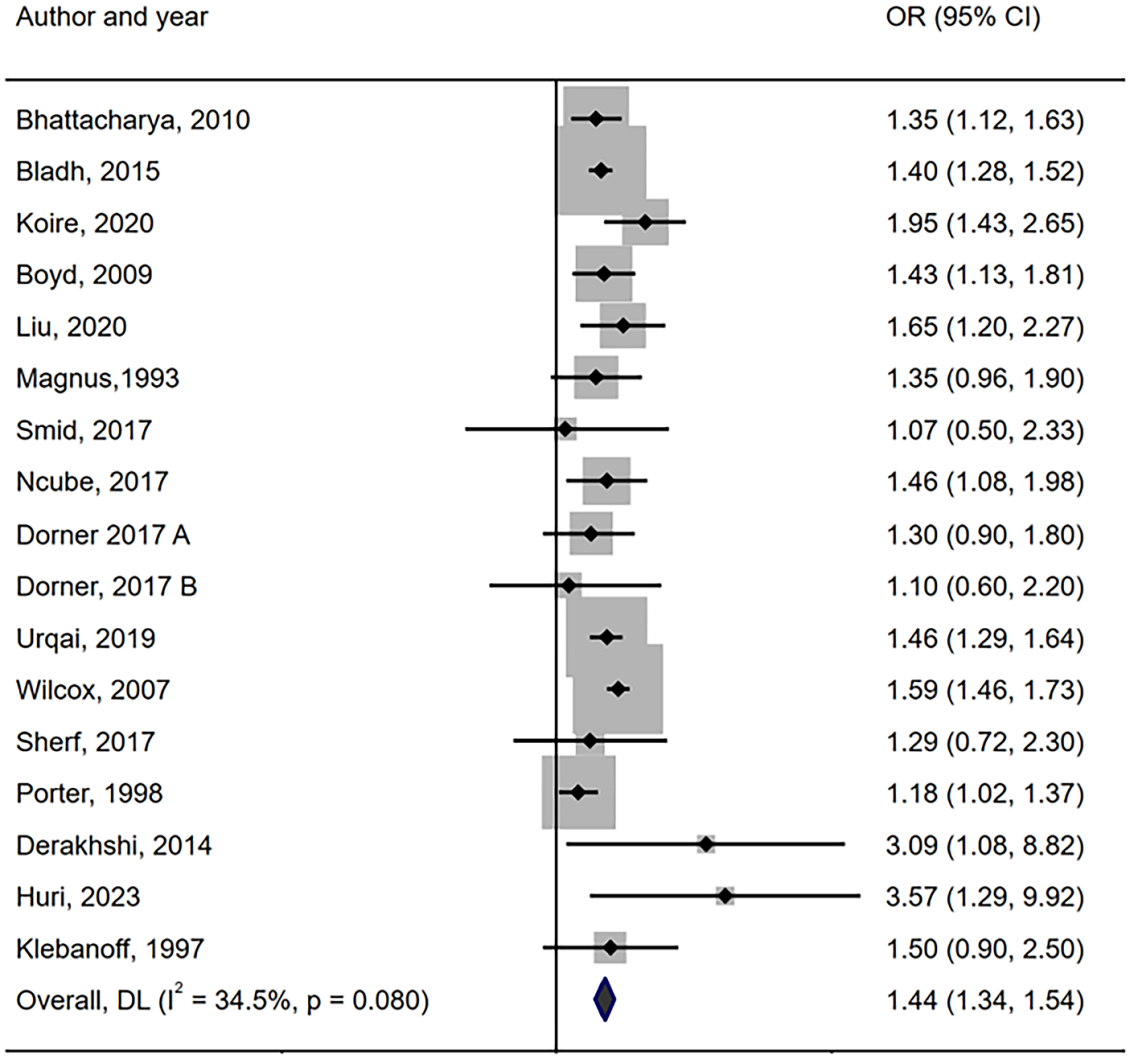
Meta‐analysis of the association between mother being born preterm and giving birth to preterm child among mothers.

The findings indicated that women who were born preterm had increased odds of giving birth to a preterm child (OR = 1.44, 95% CI: 1.34, 1.54), with a moderate level of heterogeneity (*I* ^2^ = 34.5%; Figure 2). The assessment of publication bias revealed no funnel plot asymmetry (Figure S1), with a non‐significant Egger's test (*p*‐value of 0.43).

#### 3.3.2 Subgroup analysis

To explore the cause of heterogeneity among studies reported on the relation between being born preterm and giving preterm birth, subgroup analyses were conducted based on the study designs and adjustment of confounders. The subgroup analysis based on the study designs indicated a higher estimate of the odds of preterm birth in case–control studies (OR = 3.33, 95% CI: 1.60, 6.92, *I* ^2^ = 0%), compared to cohort studies (OR = 1.43, 95% CI: 1.34, 1.52, *I* ^2^ = 27.9%) with significant between‐group heterogeneity (*p*‐value 0.02; Figure S2). An additional subgroup analysis based on whether or not adjustment of confounders was performed in the included studies (Figure S3) indicated no evidence of subgroup effect.

#### 3.3.3 Sensitivity analysis

**FIGURE 3:**
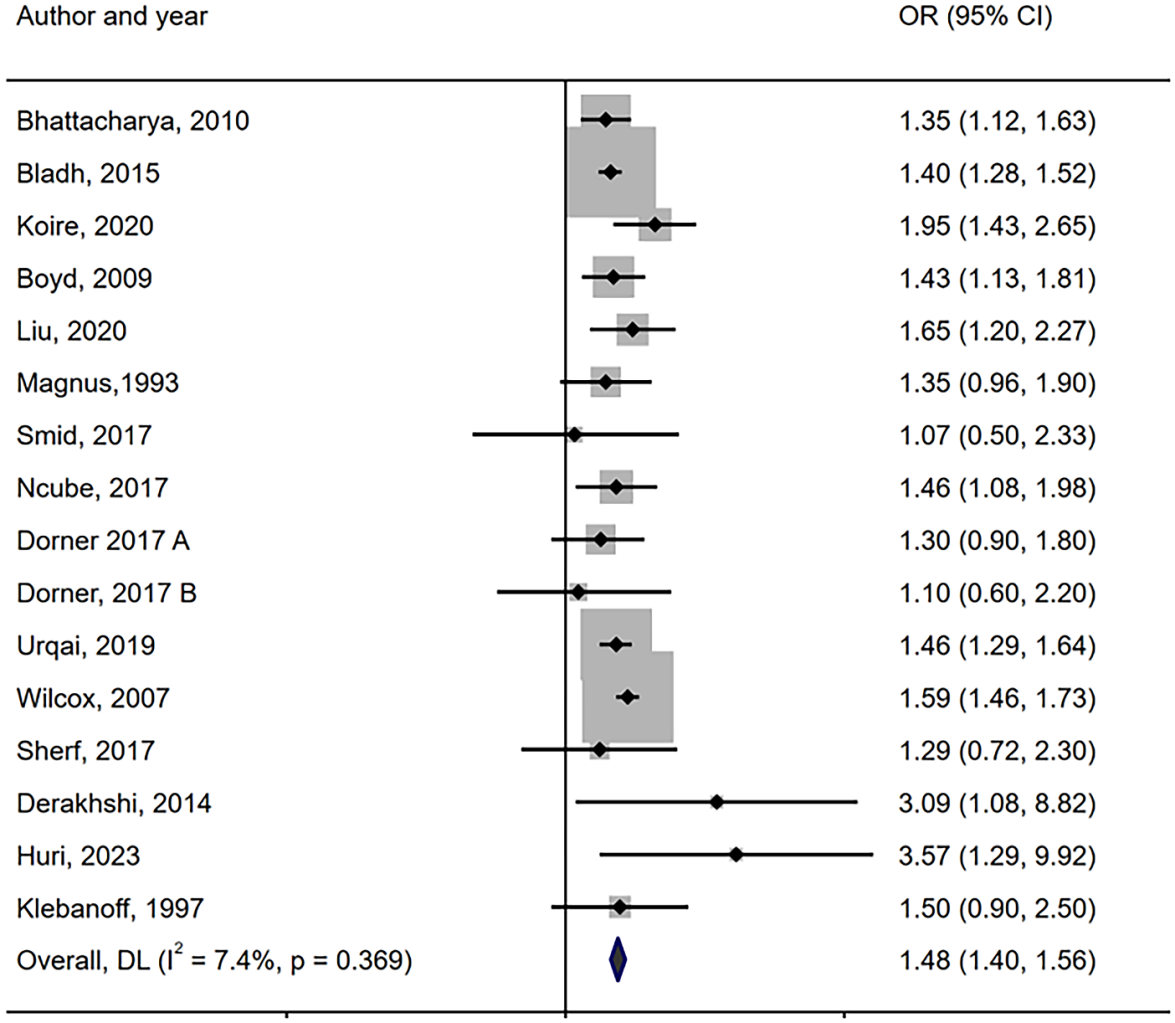
Meta‐analysis of the association between mother being born preterm and giving birth to preterm child among mothers (sensitivity analysis).

The planned sensitivity analysis, employing a ‘leave one out’ approach, was conducted by removing one outlier study^28^ contributing to a relatively large effect (OR = 1.48, 95% CI:1.40, 1.56, *I* ^2^ = 7.4%; Figure 3). However, as this effect size was not meaningfully different from the overall effect size (OR = 1.44, 95% CI: 1.34, 1.54), there is no change in overall interpretation.

Additional sensitivity analyses were performed by excluding studies published before 2000 and those that included mixed singleton and twin outcomes. The analysis of studies published after 2000 revealed no significant difference in comparison to the overall estimate of effect (OR = 1.48, 95% CI:1.41, 1.55 vs. OR = 1.44, 95% CI: 1.34, 1.54) among women who were born preterm compared to those born full‐term (Figure S4). Further sensitivity analysis by excluding studies reported on mixed sample of twin and singleton has also found almost similar effect estimate (AOR = 1.44, 95% CI:1.33, 1.57) with overall effect size (OR = 1.44, 95% CI: 1.34, 1.54; Figure S5).

#### 3.3.4 Analysis for first‐time mothers

The analysis of first‐time mother showed that women who were born preterm had a 1.49 (OR = 1.49, 95% CI: 1.41, 1.58) fold increase in odds of giving preterm birth. No heterogeneity was observed in this analysis (Figure S6). However, due to the limited number of studies included in the analysis, assessing publication biases was not possible.

#### 3.3.5 Gestational age at the mother's birth and the preterm status of the child born to the mother

The findings indicated that as the gestational age at which the mother was born decreases, the odds of giving preterm birth increases, suggesting a direct relationship between maternal gestational age at birth and that of the child.^15^, ^23^, ^28^, ^33^ For example, a cohort study conducted by Porter et al showed a 2.38 (OR = 2.38, 95% CI: 1.37, 4.16) increased odds of giving preterm birth among women who were born at the gestational age of <30 weeks compared to a 1.18 (OR = 1.18, 95% CI: 1.02, 1.37) increased odds giving preterm birth for those who were born at <37 weeks.^28^ Similar findings were reported by Urqai et al.^33^ reporting a higher odd of preterm (OR = 1.86, 95% CI: 1.31, 2.64) among women born between 24 and 31 weeks compared to those born 32–36 weeks (OR = 1.51, 95% CI; 1.34, 1.71).

#### 3.3.6 Having mother or aunt with history of giving preterm birth

Study conducted by Bhattachrya et al reported that women whose mothers gave preterm birth at any time had a higher odd of giving birth preterm (OR = 1.35, 95% CI: 1.12, 1.63).^12^ Similarly, women whose mother or aunt had history of preterm birth were likely to give birth preterm (OR = 1.34, 95% CI: 1.01, 1.74).^25^

#### 3.3.7 Having maternal siblings born preterm

The findings from five studies have indicated an increased odds (OR = 1.53, 95% CI = 1.24, 1.87) of giving birth preterm among women with a sibling born preterm compared to those with siblings born at term (Figure S7). However, there was substantial level of heterogeneity among included studies (*I* ^2^ = 74.5%). Furthermore, in one study that investigated the influence of the number of siblings born preterm, the findings indicated higher odds of preterm birth among women with two or more siblings born preterm (AOR = 1.85, 95% CI: 1.15, 2.99) compared to those who had one sibling born preterm (AOR = 1.36, 95% CI: 1.01, 1.85).^26^

#### 3.3.8 Male partner born preterm

Three studies reported the relationship between the baby's biological father (the woman's male partner) being born preterm and the woman giving birth preterm. The meta‐analysis of these studies indicated that women with a male partner born preterm had increased odds of giving birth preterm (OR = 1.12, 95% CI: 1.01, 1.25), with no heterogeneity detected among included studies (Figure S8).

#### 3.3.9 Male partners' family history of preterm birth

The relation between history of preterm birth among the extended family of the male partner and preterm birth was reported in few studies. The meta‐analysis of two studies suggested uncertain link between having a male partner with a preterm‐born sibling and the likelihood of giving preterm birth among mothers (OR = 1.05, 95% CI: 0.99–1.11, *I* ^2^ = 0%; Figure S9). Similarly, there was uncertainty in estimating whether women who had partners whose other first/second degree relatives (excluding siblings) born preterm had increased odds of giving preterm birth (OR = 0.97, 95% CI: 0.36–2.6).^34^

## 4 DISCUSSION

### 4.1 Main findings

This systematic review and meta‐analysis investigated the association between maternal and paternal family histories of preterm births and their impact on the risk of preterm birth in offspring. The overall findings revealed increased odds of preterm birth among women who were born preterm. Similarly, women whose siblings born preterm and those whose mothers/aunts born preterm were at increased odds of giving preterm birth.^25^ However, the intergenerational association of preterm birth along the paternal line revealed a less consistent association. Analysis of partner family history of preterm birth has indicated that having a partner born preterm was associated with small increase in the odds of preterm birth, while having a partner with a sibling born preterm was not associated with having a preterm birth.

### 4.2 Strengths and limitations of the study

To the best of our knowledge, this study is the first of its kind in systematically synthesising evidence on the intergenerational association of preterm births. A notable strength lies in the utilisation of predominantly larger population‐based primary studies with adequate samples that enhance the robustness of the findings of this review. Additionally, an extensive search strategy without imposing restrictions based on publication dates or geographical regions was considered as important strengths of this review. Despite these strengths, it is crucial to acknowledge certain limitations inherent in this review. One challenge in this review was the inability to differentiate between spontaneous and iatrogenic preterm births due to a lack of adequate reporting in the primary studies. While both iatrogenic and spontaneous preterm births could share common risk factors like a previous history of preterm birth,^36^ which may be influenced by genetic and epigenetic factors, iatrogenic preterm births are largely due to maternal and fetal health issues that are not exclusively explained by genetic or epigenetic factors such as hypertensive disorders, antepartum haemorrhage, fetal growth restrictions and fetal distress. Therefore, including women with iatrogenic preterm birth may obscure the true associations between family history of preterm birth and preterm birth among their biological offspring. Additionally, caution is needed when generalising the findings to women in low‐income countries, as the majority of primary studies included were sourced from higher‐income countries. Furthermore, the inclusion of a relatively small number of studies, along with substantial heterogeneity in some analyses, constitutes a limitation that requires careful consideration when interpreting the findings.

### 4.3 Interpretation of findings

The understanding of factors contributing to preterm birth remains incomplete, as around half of preterm births occur without identifiable risk factors.^37^ Recent explorations into risk factors, including genetic and epigenetic studies, have produced promising findings, hinting that a considerable portion of genetic‐epigenetic risks being transmitted through the family lineage.^38^ In concordance with this, our findings suggest that women who were born preterm and those who have sibling born preterm face an increased odds of giving preterm birth. For women with partners who were born preterm, there was only a slight increase in the odds of giving preterm birth. However, no significant association was observed among women with male partners whose siblings were born preterm or those with partners' other first/second‐degree relatives born preterm. The variation in association between preterm birth between the maternal and paternal lines may be attributed to different factors. For example, one previous study has highlighted a substantial genetic contribution to preterm birth, with heritability estimates suggesting maternal genetic involvement ranging from 15 to 40%, in contrast to a minimal contribution from the paternal genome, estimated at approximately 6%.^38^ Additionally, the increased odds of preterm birth along the maternal line may be associated with intrauterine factors specific to the maternal environment, and maternal behavioural factors that can potentially increase the likelihood of giving preterm among mothers.^38^

The findings from this systematic review indicated that mothers who were born at earlier gestational ages had a higher likelihood of giving preterm birth suggesting direct relationship between maternal gestational age at birth and gestational age of their children at birth.^15^, ^23^, ^28^, ^33^ Understanding the specific mechanisms underlying this relationship would likely require further investigation through detailed epidemiological studies and genetic analyses.

Existing evidence strongly suggests that a prior history of preterm birth is an important risk factor for recurrence of subsequent preterm births, with the risk as high as 20%.^39^ To control for the influence of previous preterm history, we conducted a subgroup analysis focusing on first‐time mothers. The findings revealed an increase in odds of giving preterm birth among first‐time mothers who were born preterm; the odds similar to overall mothers who were born preterm. The similarity may be attributed to the nature of the data utilised in our study. Most of the data from the primary studies were obtained from population registries, where women born in a specific region were longitudinally followed until they have become mothers. Although not explicitly detailed in some studies, it is plausible that a large number of women included in the registry were followed until they gave birth to their first child. This inherent characteristic of the data likely contributed to the parallel findings observed between the overall population and the subgroup of first‐time mothers. Additionally, the relatively small number of studies included in the subgroup analysis may have influenced the observed similarities.

### 4.4 Implications

The findings from this review have important implications. Women with high‐risk pregnancies often receive specific monitoring and follow‐up to prevent further complications related to pregnancy and its outcomes.^40^ Our findings underscore the critical importance of recognising maternal family history as a significant risk factor for preterm birth. Screening pregnant women for this history is essential, and should be considered to be included in antenatal care assessment. Women with a positive family history of preterm birth may benefit from closer follow‐up, and consideration should be given to referring them to clinics specialising in preterm care, when feasible.^41^ In situations where resources are limited and prioritisation is necessary for closer follow‐up, priority should be given to women with a maternal family history of preterm birth, particularly those who were born themselves at an earlier gestational age.

In this review, it was not possible to differentiate between iatrogenic and spontaneous preterm births, which affected our ability to specifically measure the association between family history and preterm births not related to medical or obstetric or fetal indications. Further studies should be conducted to differentiate between iatrogenic and spontaneous preterm births to better understand its association with family history and to design targeted interventions.

## 5 CONCLUSION

This systematic review and meta‐analysis revealed that women with a family history of preterm birth, especially those with a maternal family history, face an increased risk of giving preterm birth. This underscores the necessity of incorporating family history screening for preterm birth during pregnancy. Closer follow‐up and monitoring should be prioritised for pregnant women with a positive family history of preterm birth. Furthermore, to enhance our understanding of the effect of family history on preterm birth and to design effective specific preterm based interventions, additional studies that differentiate between spontaneous and iatrogenic preterm births are needed.

## AUTHOR CONTRIBUTIONS

AS conceptualised the study, conducted searches, extracted and analysed data, and wrote the original draft. TH contributed to study conceptualisation, screening, data extraction, analysis and writing the original draft. All authors listed contributed to conceptualisations, analysis, interpretation of findings and manuscript preparation. All authors have reviewed and approved the final version of the manuscript.

## FUNDING INFORMATION

No financial support from any funding agency were received.

## CONFLICT OF INTEREST STATEMENT

All other authors declare no competing interests.

## ETHICS APPROVAL

No ethical approval was required due to the nature of the study.

## Supporting information


Appendix S1.
Table S1.
Table S2.
Table S3.
Table S4.
Figure S1.
Figure S2.
Figure S3.
Figure S4.
Figure S5.
Figure S6.
Figure S7.
Figure S8.
Figure S9.

## Data Availability

The data that supports the findings of this study are available in the supplementary material of this article.
