# Supplementary material for: The intergenerational association of preterm birth: A systematic review and meta‐analysis
Source: BJOG. 2024 Aug 7;132(1):18–26. doi: 10.1111/1471-0528.17924 (PMC11612607; doi:10.1111/1471-0528.17924)

**Supplementary Tables**

**Appendix S1: Search terms**

**Embase Classic+Embase <1947 to 2024 January 04>**

| **No** | **Search terms** | **Results from 4 Apr 2024** |
| --- | --- | --- |
| 1 | ((preterm or premature) adj (birth* or deliver*)).mp. | 70,180 |
| 2 | inter-generation*.mp. | 617 |
| 3 | generation*.mp. | 828,025 |
| 4 | parents/ or fathers/ or mothers/ or siblings/ or spouses/ | 280,037 |
| 5 | intergeneration.mp. or exp Intergenerational Relations/ | 1,032,607 |
| 6 | 2 or 3 or 4 or 5 | 2,077,768 |
| 7 | 1 and 6 | 6,074 |
| 8 | limit 7 to (human and English language) | 4,994 |

**MEDLINE(R) <1946 to January 04, 2024>**

| **No** | **Search terms** | **Results from 4 Apr 2024** |
| --- | --- | --- |
| 1 | ((preterm or premature) adj (birth* or deliver*)).mp. | 50,898 |
| 2 | inter-generation*.mp. | 506 |
| 3 | generation*.mp. | 661,036 |
| 4 | parents/ or fathers/ or mothers/ or siblings/ or spouses/ | 168,447 |
| 5 | intergeneration.mp. or exp Intergenerational Relations/ | 4,522 |
| 6 | 2 or 3 or 4 or 5 | 829,109 |
| 7 | 1 and 6 | 2,247 |
| 8 | limit 7 to (English language and humans) | 2,019 |

**Maternity & Infant Care Database (MIDIRS) <1971 to January 04, 2024>**

| **No** | **Search terms** | **Results from 4 Apr 2024** |
| --- | --- | --- |
| 1 | ((preterm or premature) adj (birth* or deliver*)).mp. | 18,058 |
| 2 | inter-generation*.mp. | 29 |
| 3 | generation*.mp. | 2,378 |
| 4 | parent*.mp. | 26,477 |
| 5 | father*.mp. | 5,638 |
| 6 | mother*.mp. | 59,981 |
| 7 | sibling.mp. | 713 |
| 8 | intergeneration*.mp. | 462 |
| 9 | 2 or 3 or 4 or 5 or 6 or 7 or 8 | 81,693 |
| 10 | 1 and 9 | 4,097 |

**Table S1: Article excluded after full text review**

| **No** | **Article** | **Reason for exclusion** |
| --- | --- | --- |
| 1 | Castrillio SM, Rankin KM, David RJ, Collins JW, Jr. Small-for-gestational age and preterm birth across generations: a population-based study of Illinois births. Maternal and child health journal. 2014;18(10):2456-64 | Data overlap with Dorner et al, 2017. |
| 2 | McAndrew S, Chihara I, Rankin KM, Collins JW, Jr. The Relation of Maternal Birth Weight to African-American and Non-Latina White Twin Pregnancy Outcomes: A Population-Based Study. Maternal and child health journal. 2017;21(3):531-9 | Wrong population (included only twin) |
| 3 | Tocharoen, Thompson, Addy, Sargent, Best, Shoob. Intergenerational and environmental factors influencing pregnancy outcomes. Annals of epidemiology. 2000;10(7):475-6 | Wrong exposure |
| 4 | Jia Sun, Rong Yang, Hong Xian, Bin Zhang, Hualiang Lin, Pam Xaverius, Shaoping Yang, Guang-Hui Dong, Steven W. Howard, Morgan LeBaige, Anna Peng, Echu Liu, Tongzhang Zheng, Aifen Zhou & Zhengmin Qian (2022) Association between maternal family history of hypertension and preterm birth: modification by noise exposure and multivitamin intake, The Journal of Maternal-Fetal & Neonatal Medicine, 35:26, 10458-10465, DOI: [10.1080/14767058.2022.2128665](https://doi.org/10.1080/14767058.2022.2128665) | Wrong outcome. |
| 5 | Shrimali BP, Pearl M, Karasek D, Reid C, Abrams B, Mujahid M. Neighborhood Privilege, Preterm Delivery, and Related Racial/Ethnic Disparities: An Intergenerational Application of the Index of Concentration at the Extremes. Am J Epidemiol. 2020 May 5;189(5):412-421. doi: 10.1093/aje/kwz279. PMID: 31909419. | Wrong outcome |
| 6 | Swamy GK, Ostbye T, Skjaerven R. Association of preterm birth with long-term survival, reproduction, and next-generation preterm birth. JAMA. 2008 Mar 26;299(12):1429-36. doi: 10.1001/jama.299.12.1429. Erratum in: JAMA. 2008 Jul 9;300(2):170-1. PMID: 18364485. | Data overlap with Magnus et al,1993 |
| 7 | Selling KE, Carstensen J, Finnstrom O, Sydsjo G. Intergenerational effects of preterm birth and reduced intrauterine growth: a population-based study of Swedish mother-offspring pairs. BJOG : an international journal of obstetrics and gynaecology. 2006;113(4):430-40 | Data overlap with Bladh et al, 2015 |
| 8 | chulsinger C, Mednick BR, Klebanoff MA, Secher NJ, Teasdale TW, Baker RL. Delivery of preterm and small for gestational age infants across generations. Acta Psychiatrica Scandinavica, Supplement. 1993;87(370):62-6 | Wrong outcome |
| 9 | Foster HW, Wu L, Bracken MB, Semenya K, Thomas J, Thomas J. Intergenerational effects of high socioeconomic status on low birthweight and preterm birth in African Americans. J Natl Med Assoc. 2000 May;92(5):213-21. PMID: 10881470; PMCID: PMC2640563. | Wrong exposure |
| 10 | Srinivasjois RM, Gebermedhin A, Malacova E, Pereira G. Do adults born preterm deliver preterm babies? A record linkage study from Western Australia. J Matern Fetal Neonatal Med. 2022 Dec;35(25):4997-5000. doi: 10.1080/14767058.2021.1874339. Epub 2021 Feb 22. PMID: 33618594. | Wrong exposure |
| 11 | Farrant BM, White SW, Shepherd CCJ. Trends and predictors of extreme preterm birth: Western Australian population-based cohort study. PLoS One 2019; 14(3): e0214445. | Wrong exposure |
| 12 | Liu D, Lin G, Qu M, Alexander JM. Socioeconomic impacts on the intergenerational associations of preterm birth. The journal of maternal-fetal & neonatal medicine : the official journal of the European Association of Perinatal Medicine, the Federation of Asia and Oceania Perinatal Societies, the International Society of Perinatal Obstetricians. 2022;35(25):7173-8 | Date overlap with Liu D et al, 2020. |
| 13 | Emanuel I, Leisenring W, Williams MA, Kimpo C, Estee S, O'Brien W, et al. The Washington State Intergenerational Study of Birth Outcomes: methodology and some comparisons of maternal birthweight and infant birthweight and gestation in four ethnic groups. Paediatric and perinatal epidemiology. 1999;13(3):352-69. | Wrong exposure. |
| 14 | Svensson AC, Sandin S, Cnattingius S, Reilly M, Pawitan Y, Hultman CM, Lichtenstein P. Maternal effects for preterm birth: a genetic epidemiologic study of 630,000 families. Am J Epidemiol. 2009 Dec 1;170(11):1365-72. doi: 10.1093/aje/kwp328. Epub 2009 Oct 23. PMID: 19854802. | Data overlap with with Bladh et al, 2015 |
| 15 | Goldenberg RL, Andrews WW, Faye-Petersen O, Cliver S, Goepfert AR, Hauth JC. The Alabama Preterm Birth Project: placental histology in recurrent spontaneous and indicated preterm birth. Am J Obstet Gynecol. 2006 Sep;195(3):792-6. doi: 10.1016/j.ajog.2006.05.050. Epub 2006 Jul 17. PMID: 16846583. | Wrong exposure |
| 16 | Emanuel I, Leisenring W, Williams MA, Kimpo C, Estee S, O'Brien W, Hale CB. The Washington State Intergenerational Study of Birth Outcomes: methodology and some comparisons of maternal birthweight and infant birthweight and gestation in four ethnic groups. Paediatr Perinat Epidemiol. 1999 Jul;13(3):352-69. doi: 10.1046/j.1365-3016.1999.00184.x. PMID: 10440054. | Wrong exposure |
| 17 | Hennessy E, Alberman E. Intergenerational influences affecting birth outcome. II. Preterm delivery and gestational age in the children of the 1958 British birth cohort. Paediatr Perinat Epidemiol. 1998 Jul;12 Suppl 1:61-75. doi: 10.1046/j.1365-3016.1998.0120s1061.x. PMID: 9690274. | Wrong exposure |
| 18 | Collins JW, Rankin KM, David RJ. Low birth weight across generations: the effect of economic environment. Matern Child Health J. 2011 May;15(4):438-45. doi: 10.1007/s10995-010-0603-x. PMID: 20390329. | Wrong exposure |
| 19 | Collins, J.W., et al., *Early and Late Preterm Birth Rates Among US-Born Urban Women: The Effect of Men's Lifelong Class Status.* Maternal & Child Health Journal, 2019. **23**(12): p. 1621-1626. | Wrong exposure |
| 20 | Drukker L, Haklai Z, Ben-Yair Schlesinger M, Bas-Lando M, Gordon ES, Samueloff A, Schimmel MS, Grisaru-Granovsky S. "The next-generation": Long-term reproductive outcome of adults born at a very low birth weight. Early Hum Dev. 2018 Jan;116:76-80. doi: 10.1016/j.earlhumdev.2017.11.011. Epub 2017 Nov 29. PMID: 29197251. | Wrong exposure |

**Table S2: Characteristics of studies included in the systematic review and meta-analysis.**

| **Authors and year** | **Country** | **Study design** | **Inclusion** | **Exclusion** | **Sample size** | **Exposure** | **Outcome** | **Effect size** | **Confounders adjusted** |
| --- | --- | --- | --- | --- | --- | --- | --- | --- | --- |
| Bhattacharya, S., et al. (2010) | UK | Cohort | Singleton deliveries occurring in mother–daughter pairs (first born), spontaneous preterm birth | Multiple pregnancy | Mothers (n=11,576) | Mother with maternal history of preterm.  Moher born preterm | Child born preterm | a, Preterm among mother with maternal history of any preterm birth:  (OR 1.35, 95% CI 1.12–1.63),  b1, Preterm among mother who were born preterm  (OR=1.49, 95%CI: 1.12–1.99),  b2, Preterm among nulliparous mother born preterm  (OR=1.60, 95%CI= 1.16, 2.21) | Adjusted for age at delivery, social class, smoking status, BMI, multi parity, previous miscarriage, previous preterm delivery, HDP, preterm delivery |
| Bladh, M., et al. (2015) | Sweden | Cohort | Only preterm status of firstborn children was included in the analysis (first born) | Missing values on birth weight and/or gestational age, extremely high birth weight, extremely low birthweights, higher order above twin were excluded | Mothers (n=264,794) | Singleton mother born preterm herself. | Child born preterm | Preterm born to mother born preterm, (OR=1.40, 95% CI:1.28,1.52), | Adjusted for grandmother age, grandmother marital status, origin (nordic vs not nordic), grandmother educational level, grandmother parity, mother age, mother marital status, mother educational level, BMI, nicotine use, twin birth child, SGA |
| Liu, D et al. 2020 | USA | Cohort | All singleton births between 2010 and 2018 from and their mothers mother born from 1995 to 2005. Mother with BW =>500 g and  gestational age between 22 – 24 wks included. | Maternal age at delivery<14 years old was excluded | Mothers (n=5,118) | Mother born preterm  Mother’s sibling born preterm | Child born preterm | Preterm born to mother born preterm,  (OR =1.65, 95%CI: 1.20, 2.27).  Preterm born to mother with sibling born preterm, OR=1.47 (1.13, 1.91),  Preterm born to mother with one sibling born preterm, OR=1.36, 95%CI= 1.01, 1.85)  Preterm born to mother with 2+ sibling born preterm,  OR=1.85, 95%CI=1.15, 2.99) | Adjusted for gestational hypertension or gestational diabetes, mother used tobacco during pregnancy, mother received C-section, grand-  mother was African American, and grandmother lived in the urban area. |
| Ncube, C.N. (2017) | USA | Cohort | Only singleton first born infants were included | Infants with congenital anomalies, non-black or white, mothers who were Hispanic, infants with GA<20 weeks, birth record with missing on GA, race | Mothers (n=6592) | Mother born preterm | Child born preterm | Preterm born to mother born preterm, (OR=1.46, 95%CI: 1.08, 1.98) | Adjusted for race, maternal age, marital status, educational attainment, health insurance, intergenerational neighbourhood racial composition and poverty |
| Sherf, et al. (2016) | Israel | Cohort | Only singleton or twin were included | More than twin delivery and children with birth weight of<500 gm. | Mothers  (n=1616) | Mother born preterm | Child born preterm | Preterm born to mother born preterm, (OR 1.29, 95% CI :0.72, 2.30) | Adjusted for Maternal age, parity, pre-eclampsia |
| Dorner, et al. (2017) **A** | USA | Cohort | Only singleton included (African-American women) | Women with multiple gestations | Mothers  (n=37,791) | Mother born preterm at gestational age of <30 wks. | Child born preterm | Preterm born to mother born preterm:  Extreme preterm (OR=2.31, 95%CI: 1.2, 4.3)  Modestly early preterm (OR=1.5, 95%CI: 0.9, 2.7)  **Late preterm (OR=1.3, 95%CI: 0.9, 1.8)** | Adjusted for maternal age, parity, education, prenatal care, marital status, cigarette smoking |
| Dorner, et al. (2017) **A** | USA | Cohort | only singleton included (African-American women) | Women with multiple gestations | Mothers  (n=37,791) | Mother born at gestational age of 30-33 weeks. | Child born preterm | Preterm born to mother born preterm:  Extreme preterm (OR=1.9, 95%CI: 1.3.-2.6)  Modestly early preterm (OR=1.3, 95%CI: 0.9, 1.7)  Late preterm (OR= 1.2, 95%CI: 1.0, 1.5) | Adjusted for maternal age, parity, education, prenatal care, marital status, cigarette smoking |
| Dorner, et al. (2017) **A** | USA | Cohort | Only singleton included (African-American women) | Women with multiple gestations | Mothers  (n=37,791) | Mother born preterm at gestational age of 34-36wks | Child born preterm | Preterm born to mother born preterm:  Extreme preterm  (OR= 1.0, 95%CI: 0.8, 1.4)  Modestly early preterm (OR= 1.1, 95%CI: 0.9, 1.3)  Late preterm  (OR=1.1, 95%CI: 1.0, 1.2) | Adjusted for maternal age, parity, education, prenatal care, marital status, cigarette smoking |
| Dorner, et al. (2017) **B** | USA | Cohort | Only singleton included (non- Latina white) | Women with multiple gestations | Mothers (n=134,362 | Mother born preterm at gestational age of <30 weeks. | Child born preterm | Preterm born to mother born preterm:  Extreme preterm (OR=4.0, 95%CI: 1.2, 12.6)  Modestly early preterm (OR=3.1, 95%CI :1.2, 7.7)  **Late preterm (OR =1.1 95%CI: 0.6, 2.2)** | Adjusted for maternal age, parity, education, prenatal care, marital status, cigarette smoking |
| Dorner, et al. (2017) **B** | USA | Cohort | Only singleton included (non- Latina white) | Women with multiple gestations | Mothers  (n=134,362 | Mother born preterm at gestational age of 30-33 weeks. | Child born preterm | Preterm born to mother born preterm:  Extreme preterm (OR=1.1, 95%CI: 0.5. 2.4)  Modestly early preterm (OR=1.6, 95%CI: 1.0, 2.5)  Late preterm (OR=1.1, 95%CI: 0.8, 1.4) | Adjusted for maternal age, parity, education, prenatal care, marital status, cigarette smoking |
| Dorner, et al. (2017) **B** | USA | Cohort | Only singleton included (non- Latina white) | Women with multiple gestations | Mothers  (n=134,362) | Mother born preterm at gestational age of 34-36wks. | Child born preterm | Preterm born to mother born preterm:  Extreme preterm: (OR=1.8, 95%CI: 1.3, 2.5)  Modestly early preterm (OR=1.1, 95%CI :0.9, 1.5) Late preterm (OR=1.2, 95%CI: 1.0, 1.3) | Adjusted for maternal age, parity, education, prenatal care, marital status, cigarette smoking |
| Urquia, et al. (2019) | Canada | Cohort | Women born singleton in a Manitoba | Extreme maternal and child GAs (<24 or >42 wks), multiple pregnancy (both in mother and child), missing GAs. | Mothers (n=39,573) | Mother born preterm | Child born preterm | Preterm born to mother born preterm:  Mother born GA (24-36 weeks):  (OR=1.46, 95%CI: 1.29, 1.64)  Mother born very preterm (24-31 weeks):  (OR= 1.86, 95%CI: 1.31, 2.64)  Mother born moderate preterm (32—36 weeks) (OR=1.51, 95%CI; 1.34, 1.71) | Not adjusted or effect size calculated from raw data. |
| Huri, et al. (2023) | Italy | Case control study | Only women born singleton infants were included | multiple  pregnancies, women with iatrogenic preterm deliveries (for different indications), and with uncertain gestational age were excluded | Mothers  (n= 354) | a, Mother born preterm  b, Mother’s sibling born preterm  c, Mother’s other first/second degree relatives born preterm  d, Mother’s partner born preterm  e, Mother’s partner sibling born preterm. f, Mother’s partner other first/ second degree relatives born preterm. | Child born preterm | Preterm birth to:  a, Mother born preterm:  (OR=3.57, 95%C: 1.29, 9.92),  b, Mother who have siblings born preterm (OR=3.18, 95%CI:1.31–7.7),  c, Mother who have other first/second degree relatives born preterm  (OR= 1.56, 95%CI: 0.78–3.12)  d, Mother with partner born preterm  (OR= 0.72, 95%CI: 0.32–1.65),  e, Mother who have partner’s sibling born preterm (OR= 0.86, 95%CI: 0.27–2.73).  f, Mother who have partners other first/second degree relatives born preterm (OR=0.97, 95%CI: 0.36–2.6) | Confounder were controlled by matching cases and controls for maternal characteristics such as ethnicity, marital status and maternal education level |
| Koire et al. (2021) | USA | Cohort | Only women born singleton infants were included. Spontaneous preterm | multiple gestations, and  women with preterm due to different possible indications | Mothers  (n=23,816) | a, Mother’s sister born preterm.  b, Mother born preterm | Child born birth | **Preterm birth among (overall sample**):  a, Mother whose sisters born preterm  (OR=1.81, 95%CI: 1.43, 2.29)  b, Mother who were themselves born preterm  (OR= 1.95, 95%CI: 1.43, 2.65)  **Preterm birth among (nulli parous sample):**  a, Mother whose sisters born preterm  (OR=2.42, 95%CI: 1.44, 4.07)  b, Mother who were themselves born preterm  (OR= 1.56, 95%CI: 1.06, 2.29) | Not adjusted or effect size calculated from raw data. |
| Derakhsh et al.2014 | Iran | Case control study | Birth occurs between 20 weeks to 42 weeks of gestation regardless of singleton or multiple. | - | Mothers  (n=585) | Mother born preterm | Child born birth | Mother born preterm:  (OR= 3.09, 95%CI:1.08, 8.82) | Not adjusted or effect size calculated from raw data. |
| Porter, et al. (1998) | USA | Cohort | Only women gave birth of singleton were included | All non-white women, and women with preterm due to induction were excluded | Mothers  (n=4186) | a, Mother born preterm at gestational age <37 wks. b, Mother born preterm at gestational age <30 wks. | Child born birth | Preterm born to mother born preterm:  a, Mother born<37 wks.  (OR=1.18,95%CI:1.02,1.37)  b, Mother born GA<30 wks. (OR=2.38,95%CI:1.37,4.16) | Not adjusted or effect size calculated from raw data. |
| Magnus, e al. (1993) | Norway | Cohort | Only singleton first born who survived the first 4 weeks of life (the neonatal period were included) | multiple birth (both for mother and child) and births with GA<28 and 43 weeks were excluded | Mother (n=5385) | Mother born preterm. | Child born birth | Mother born preterm:  (OR=1.35, 95%CI: 0.96, 1.90) | Not adjusted or effect size calculated from raw data. |
| Smid, et al. (2017) | USA | Cohort | All singleton delivery white mothers were included (first born) | non first born, multifetal gestation, infants with congenital aneuploidy, infant’s gestational age at delivery <20 weeks, or the birthweight was<500 g | Mothers (n=50209) | a, Non-Hispanic White mother born preterm at gestational age 34-36 wks. b, Non-Hispanic White mother born preterm at gestational age<34 wks. | Child born birth | **Preterm born to:**  a*, Mother born between 34-36 weeks*:  i, Mother gave birth to infant <34 wks  (OR=1.28, 95%CI: 0.71,2.31),  ii, Mother gave birth to preterm born 34-36 weeks (OR =1.03, 95%CI: 0.68,1.54)  *b, Mother born preterm<34 weeks:*  i, Mother gave birth to preterm infant <34 weeks  (OR= 2.02, 95%CI: 0.81, 5.02),  ii, Mother gave birth to preterm infant 34-36 weeks  **(OR= 1.07, 95%CI:0.50, 2.33)** | Adjusted for maternal education, maternal age, smoking, drug/alcohol use, and infant gender. |
| Boyd et al. (2009) | Denmark | Cohort | women delivered singletons with known gestational age were included | twins and triplets excluded | Mother  (n=1,487,159) | a, Mother born preterm.  b, Mother’s sibling from the same mother born preterm.  c, Mother’s partner born preterm  d, Mother’s partner sibling born preterm | Child born preterm | Preterm birth among:  a, Mother born preterm  OR=1.43, 95%CI= 1.13, 1.81)  b, Mother’s sibling from the same mother born preterm OR=1.63, 95%CI= 1.55, 1.72)  c, women whose male partner born preterm (OR=1.17, 95%CI: 0.82, 1.66),  d, women whose male partner’s sibling born preterm (OR=1.05, 95%CI: 0.99, 1.11) | Not adjusted or effect size calculated from raw data. |
| Wilcox, et al. (2008) | Norway | Cohort | Included only parents and offsprings born singleton. Only first birth offsprings were included | All person with pre-eclampsia (both parents and offsprings) were excluded | Mother (n=191,282) | Mother born preterm | Child born preterm | Mothers born preterm:  (OR=1.59 (95% CI: 1.46, 1.73).  Partner born preterm  (OR= 1.113, 95% CI: 1.01, 1.27)  Preterm born to mother born early preterm (<35 weeks):  (OR= 1.89, 95%CI: 1.53, 2.33)  Preterm born to father born early preterm (<35 weeks):  OR=1.06, 95%CI: 0.77, 1.46) | Not adjusted or effect size calculated from raw data. |
| Klebanoff, et al. (1997) | Denmark | Cohort | Parous women born in the 1959 to 1961 were included in the study. | Women with uncertain or unknown dates of the LMP, those who born twin and women whose own birth weights were >90th percentile for gestational age were excluded. | Mother (n= 2029) | Mother born preterm | Child born preterm | Mother born preterm:  (OR=1.5, 95%CI= 0.9–2.5) | Adjusted for maternal residence, birth order, socioeconomic status at 1 year of age, education, adult height and weight, smoking during pregnancy, employment, parity, certainty of gestational age in generation 3, and grandmaternal height. |

**Note: Effect sizes in bold font were chosen from studies reporting multiple effect sizes for inclusion in the meta-analysis on the association between mothers born preterm and giving birth to preterm children.**

**Quality of studies included in the Review**

**Table S3: Quality of included Cohort studies**

| **Authors and year** | **Q1: were the two-group recruited from the same population** | **Q2: were exposure defined similarly among both group** | **Q3: were exposure measured in valid and reliable way** | **Q4: were confounder identified** | **Q5: Were strategies to deal with confounder stated** | **Q6: were participants free of the outcome at the start of the study** | **Q7: were outcomes measured in a valid and reliable way** | **Q8: was follow up time reported and sufficient for the outcome to occur** | **Q9: Was follow up complete, and if not reason to loss to follow up stated** | **Q10: were strategies to address incomplete follow up utilized** | **Q11: was appropriate statistical analysis used?** | **Total** |
| --- | --- | --- | --- | --- | --- | --- | --- | --- | --- | --- | --- | --- |
| Bhattacharya, (2010) | yes | yes | no | yes | yes | yes | yes | yes | no | no | yes | 8 |
| Bladh, M., et al. (2015) | yes | unclear | yes | yes | yes | yes | unclear | yes | no | no | yes | 7 |
| Liu, D., et al. (2022) | yes | unclear | yes | yes | yes | yes | unclear | yes | no | no | yes | 7 |
| Ncube, C.N. (2017) | yes | unclear | yes | yes | yes | yes | unclear | yes | no | no | yes | 7 |
| Y. Sherf, E. 2016 | yes | unclear | yes | yes | yes | yes | unclear | yes | no | unclear | yes | 7 |
| R. A. Dorner, K.2017 | yes | no | no | yes | yes | yes | yes | yes | no | yes | yes | 8 |
| Urquia, M.L. 2019 | yes | yes | yes | yes | no | yes | unclear | yes | no | no | yes | 7 |
| Koire A, 2021 | yes | no | no | yes | yes | yes | unclear | yes | yes | n/a | yes | 7 |
| MD T.Flint Porter 1998 | yes | unclear | no | yes | no | yes | unclear | yes | no | no | yes | 5 |
| P. Magnus, 1993 | yes | yes | yes | yes | yes | yes | yes | yes | no | no | yes | 9 |
| Marcela, 2017 | yes | yes | yes | yes | yes | yes | yes | no | no | no | yes | 8 |
| Boyd HA, 2009 | yes | no | yes | yes | no | yes | yes | yes | no | no | yes | 7 |
| Wilcox AJ, 2008 | yes | yes | yes | yes | no | yes | yes | yes | no | no | yes | 8 |
| Klebanoff, 1997 | yes | yes | yes | yes | yes | yes | yes | yes | no | no | yes | 9 |

**n/a: not applicable**

**Table S4: Quality of included case-control studies**

| **Authors and year** | **Q1: were the two-group comparable?** | **Q2: were cases and controls matched appropriately?** | **Q3: were the same criteria used for identification of cases and controls?** | **Q4: Was exposure measured in a standard, valid and reliable way?** | **Q5: Was exposure measured in the same way for cases and controls?** | **Q6: Were confounding factors identified?** | **Q7: Were strategies to deal with confounding factors stated?** | **Q8: Were outcomes assessed in a standard, valid and reliable way for cases and controls?** | **Q9: Was the exposure period of interest long enough to be meaningful?** | **Q10: Was appropriate statistical analysis used?** | **Total** |
| --- | --- | --- | --- | --- | --- | --- | --- | --- | --- | --- | --- |
| M. Huri, N., 2023 | Yes | yes | yes | no | yes | yes | yes | yes | yes | yes | 9 |
| Derakhshi B 2014 | Yes | yes | yes | unclear | yes | yes | no | unclear | yes | yes | 7 |

**Supplementary figures**

**Figure S1: Funnel plot for studies reported on the association between being born preterm and giving birth of preterm among mothers**


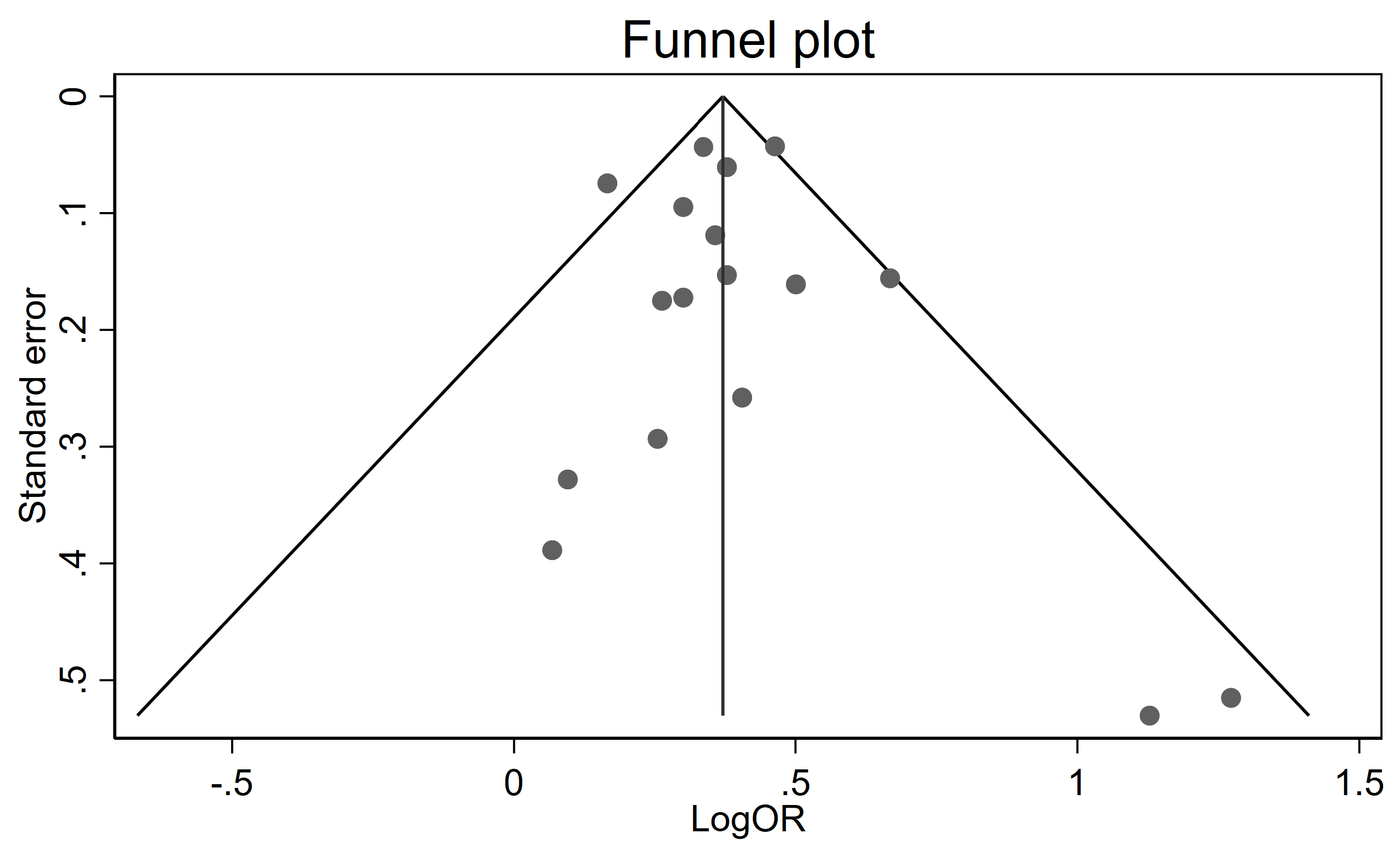


**Figure S2: Meta-analysis of the association between mother being born preterm and giving birth to preterm child among mothers (subgroup analysis by study design)**


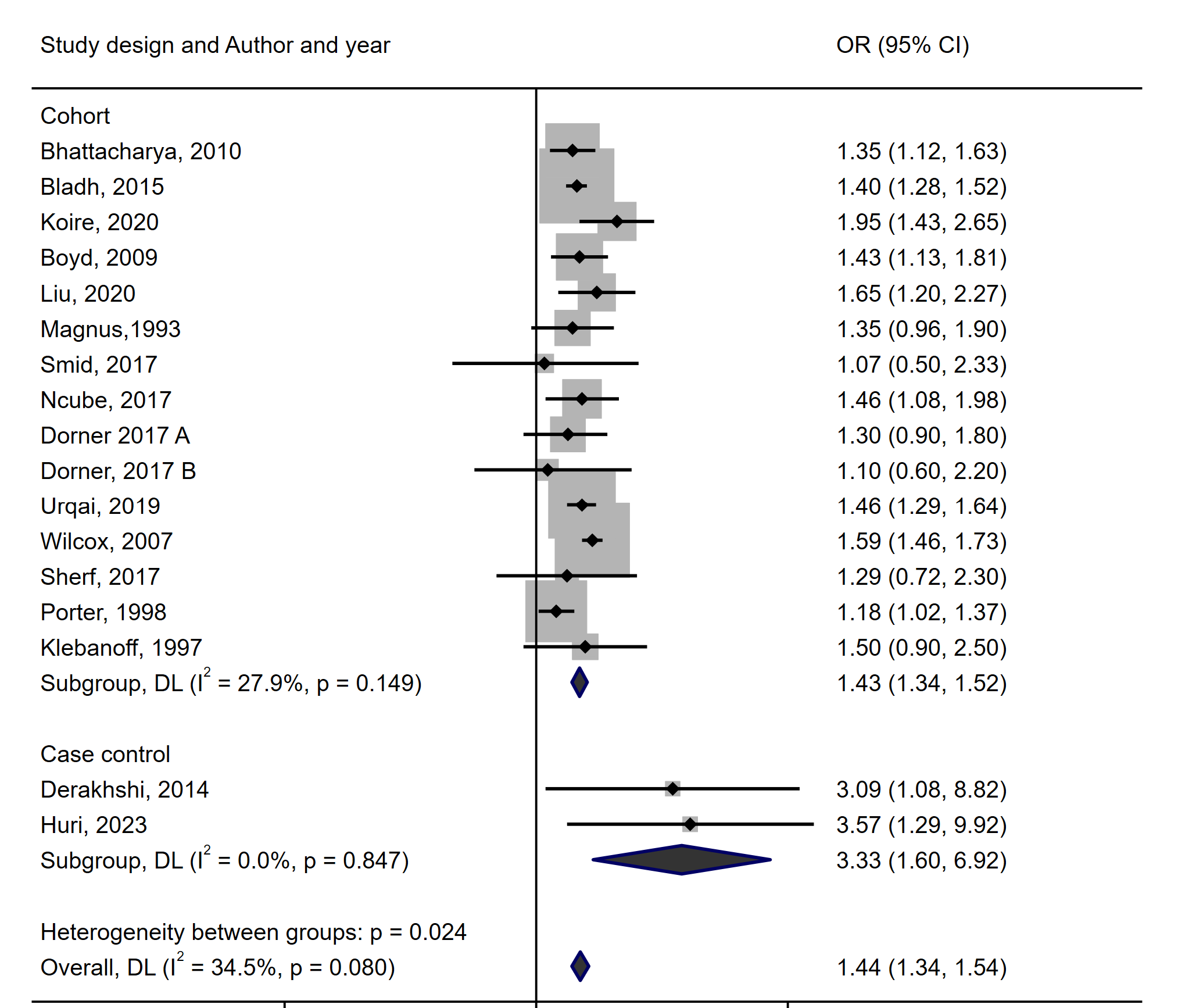


**Figure S3: Meta-analysis of the association between mother being born preterm and giving birth to preterm child among mothers (subgroup analysis by adjustment of confounders)**

**
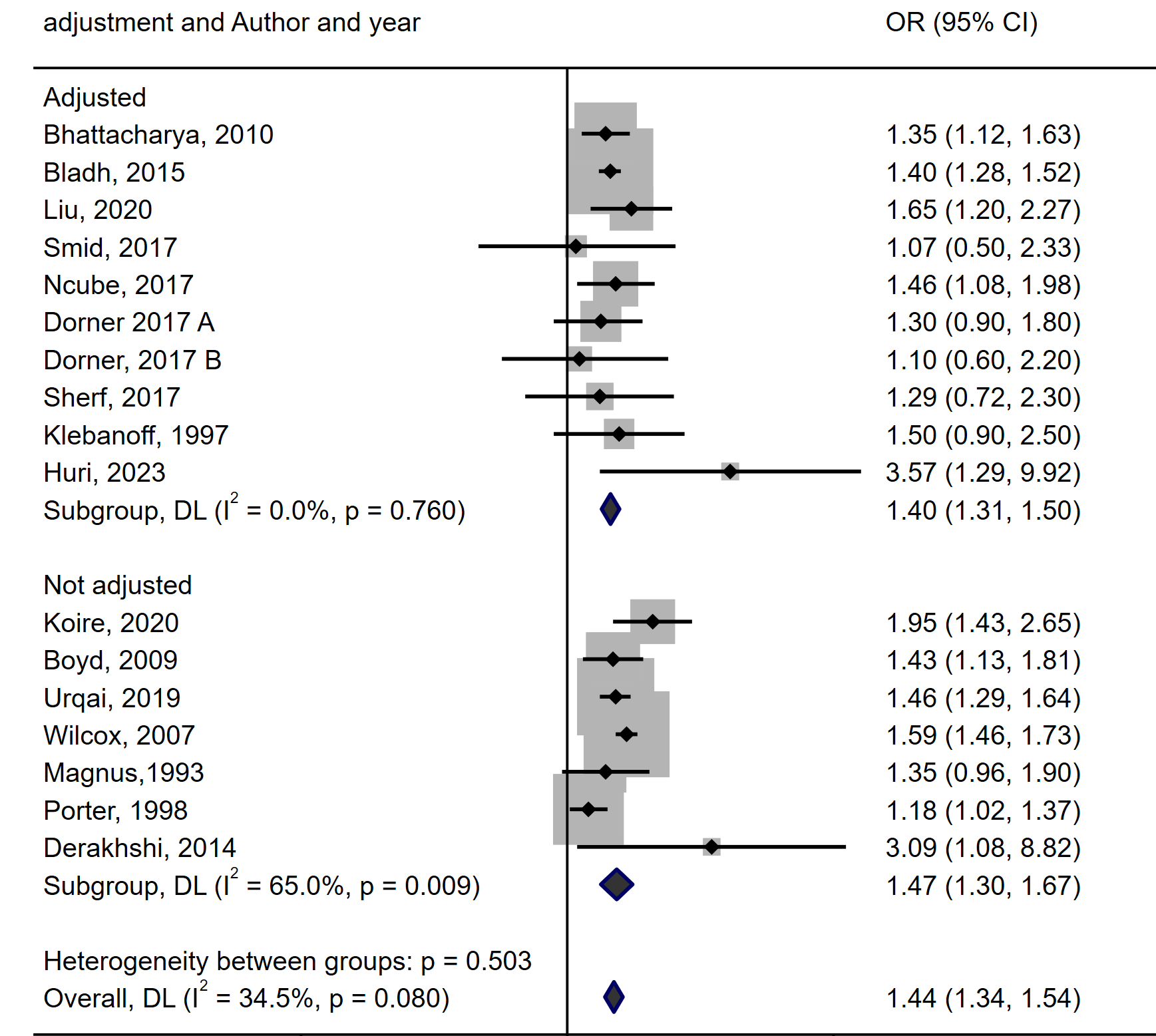
**

**Figure S4: Meta-analysis of the association between mother being born preterm and giving birth to preterm child among mothers (Sensitivity analysis by removing studies published before 2000)**

**
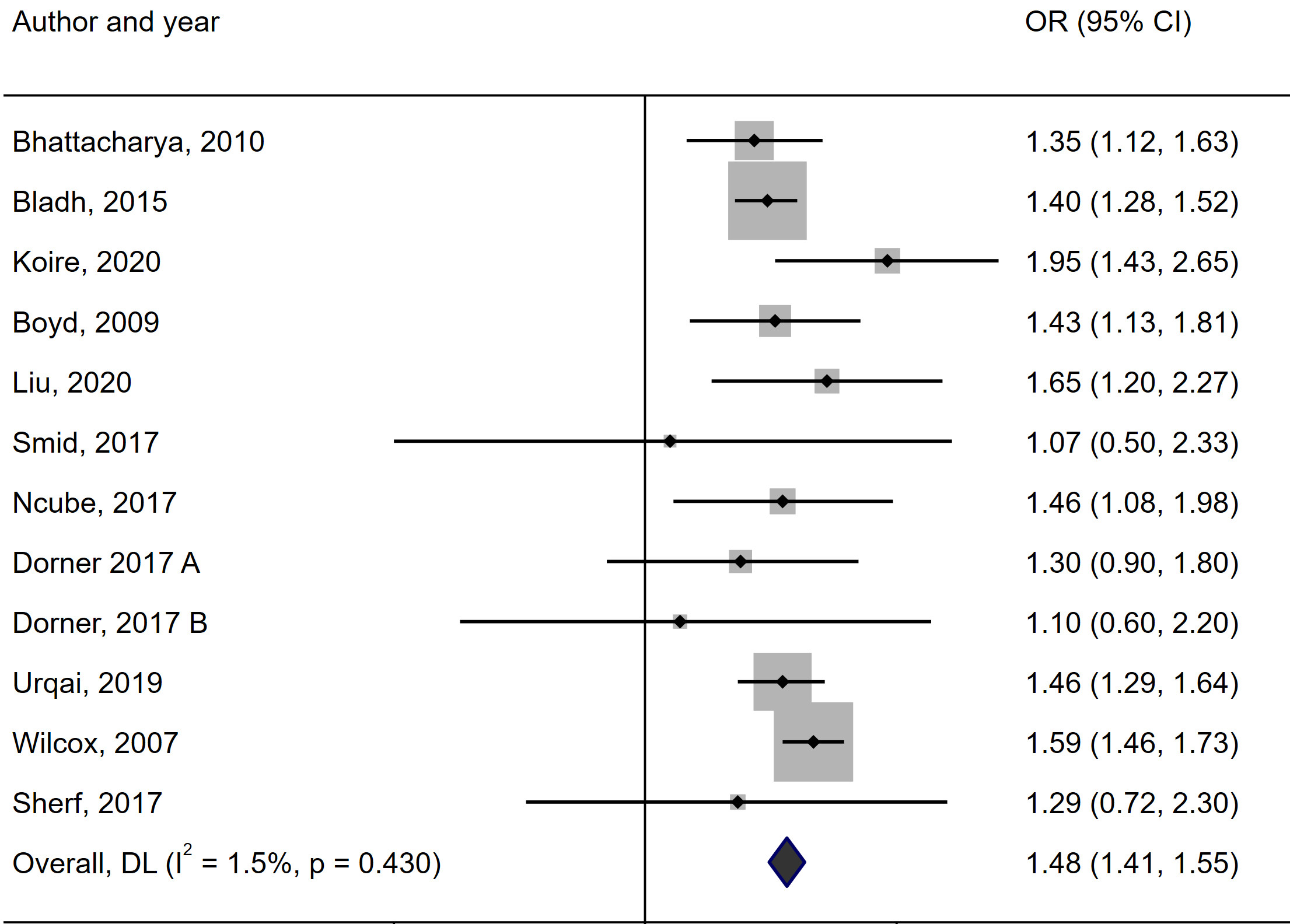
**

**Figure S5: Meta-analysis of the association between mother being born preterm and giving birth to preterm child among mothers (Sensitivity analysis by removing studies reported on mixed singleton and twin outcomes).**

**
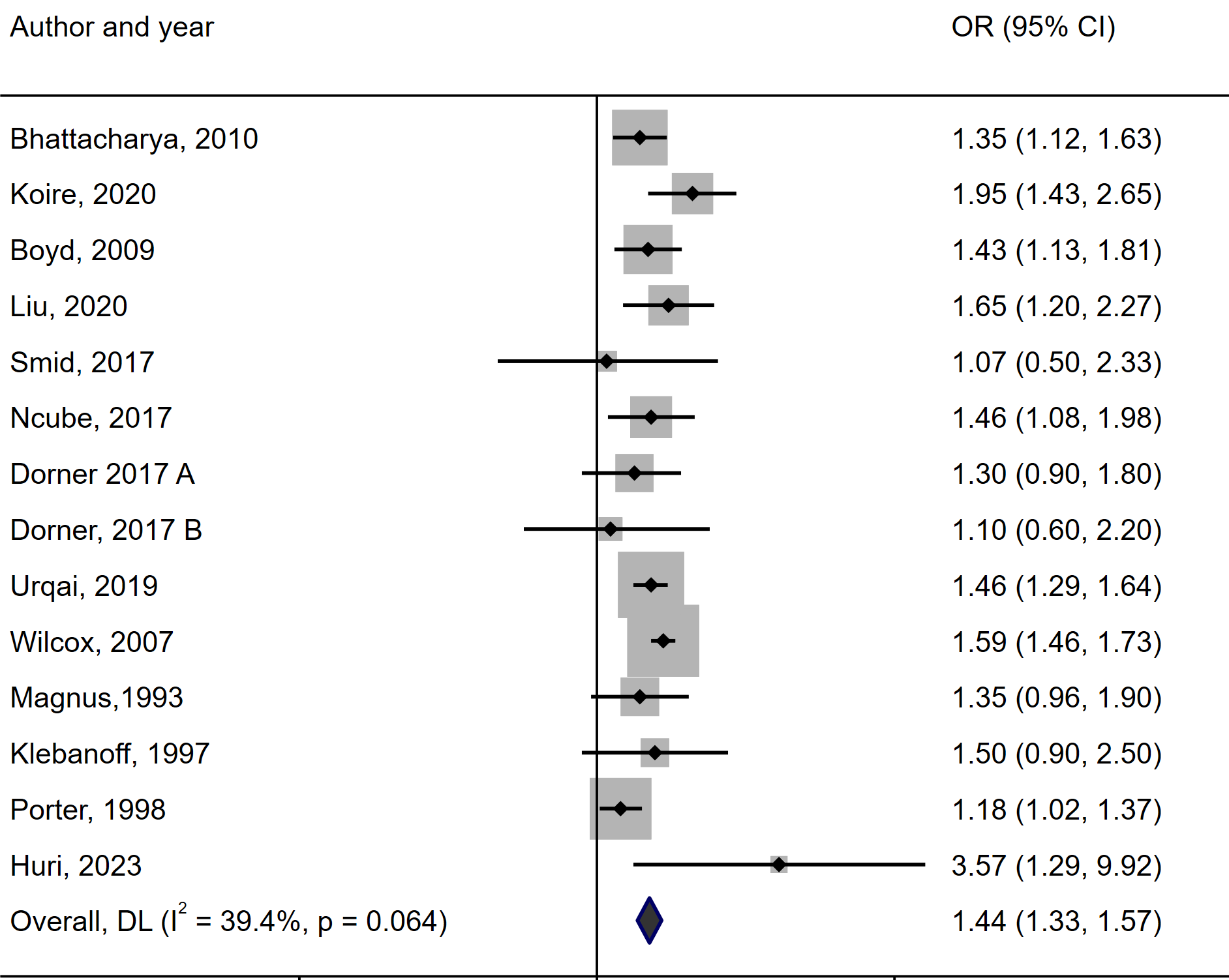
**

**Figure S6: Meta-analysis of the association between mother being born preterm and giving birth to preterm child among mothers (first time mother)**


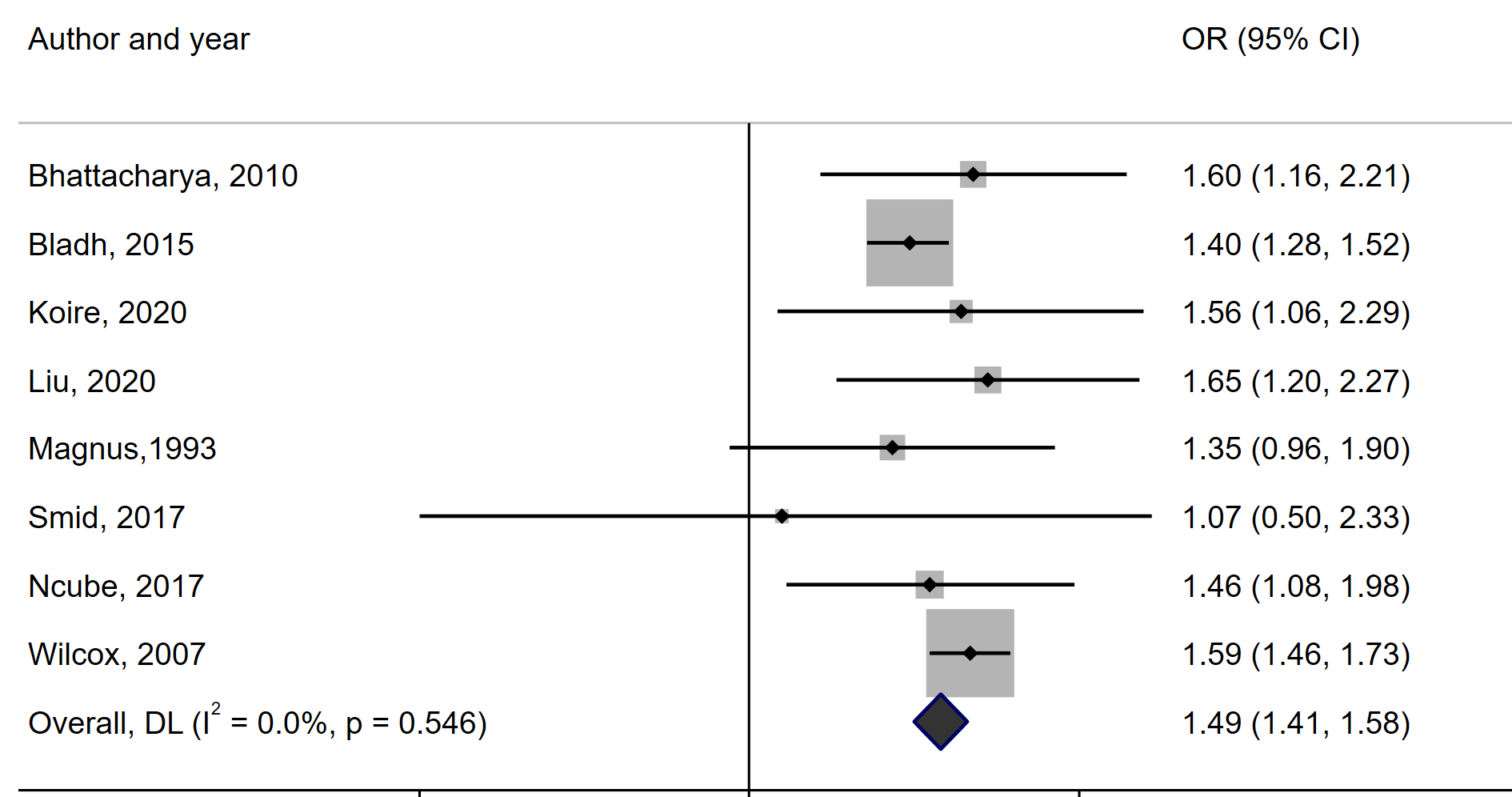


**Figure S7: Meta-analysis of the association between having maternal sibling born preterm and giving birth to preterm child among mothers**


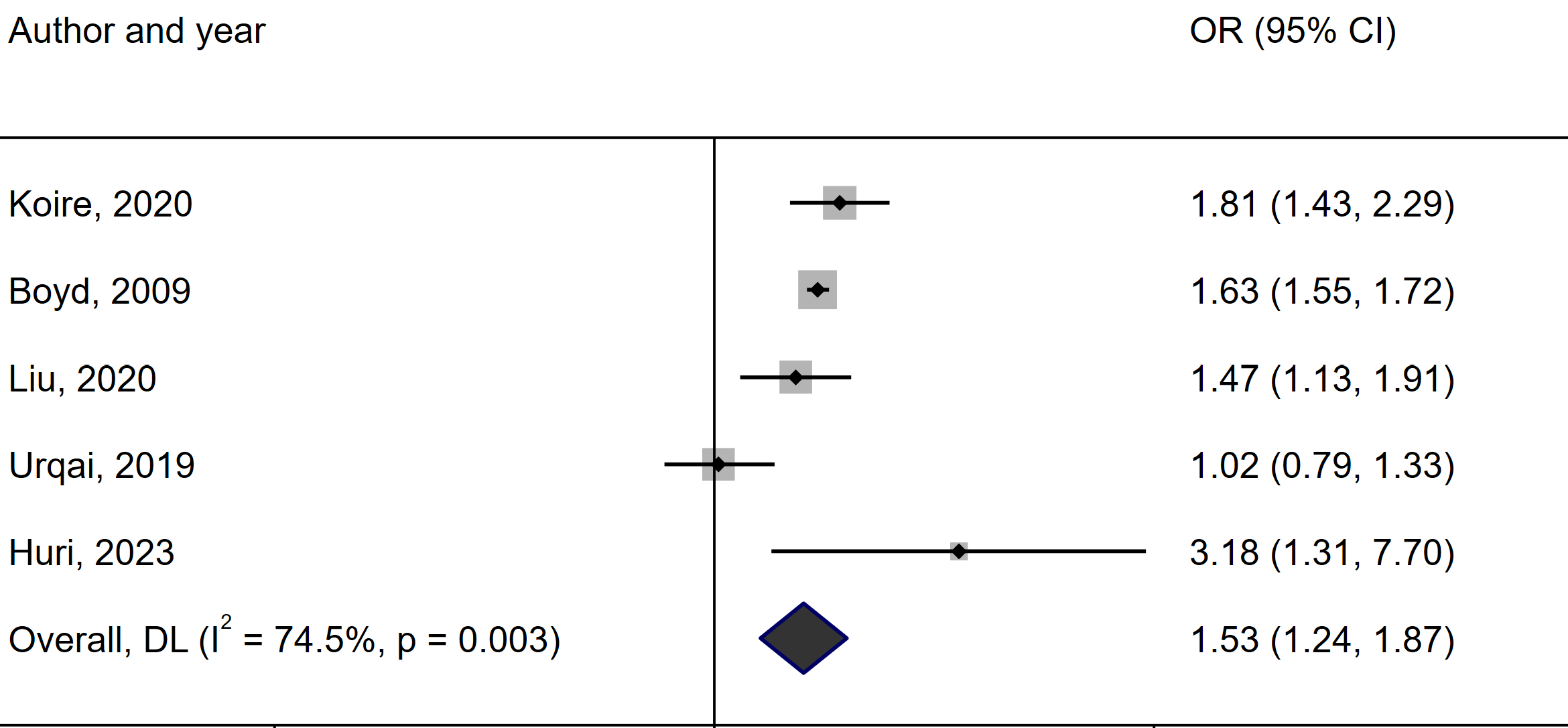


**Figure S8: Meta-analysis of the association between having partner being born preterm and giving birth to preterm child among mothers**


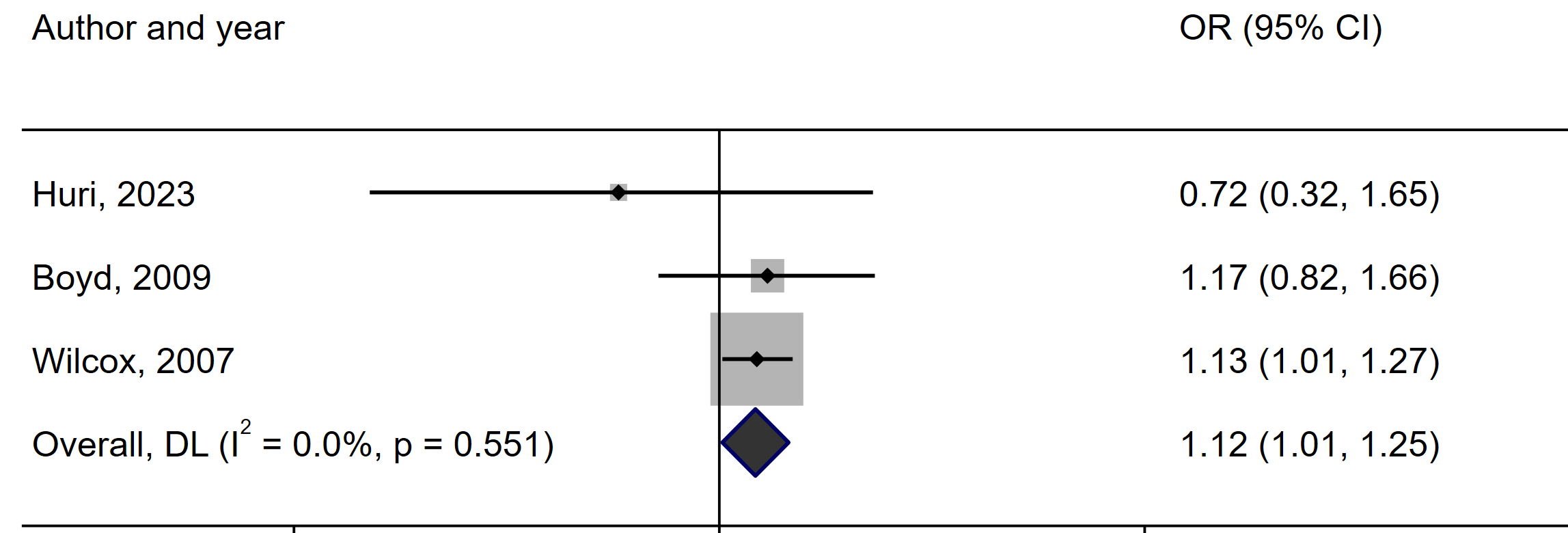


**Figure S9: Meta-analysis of the association between having partner with sibling born preterm and giving birth to preterm child among mothers**


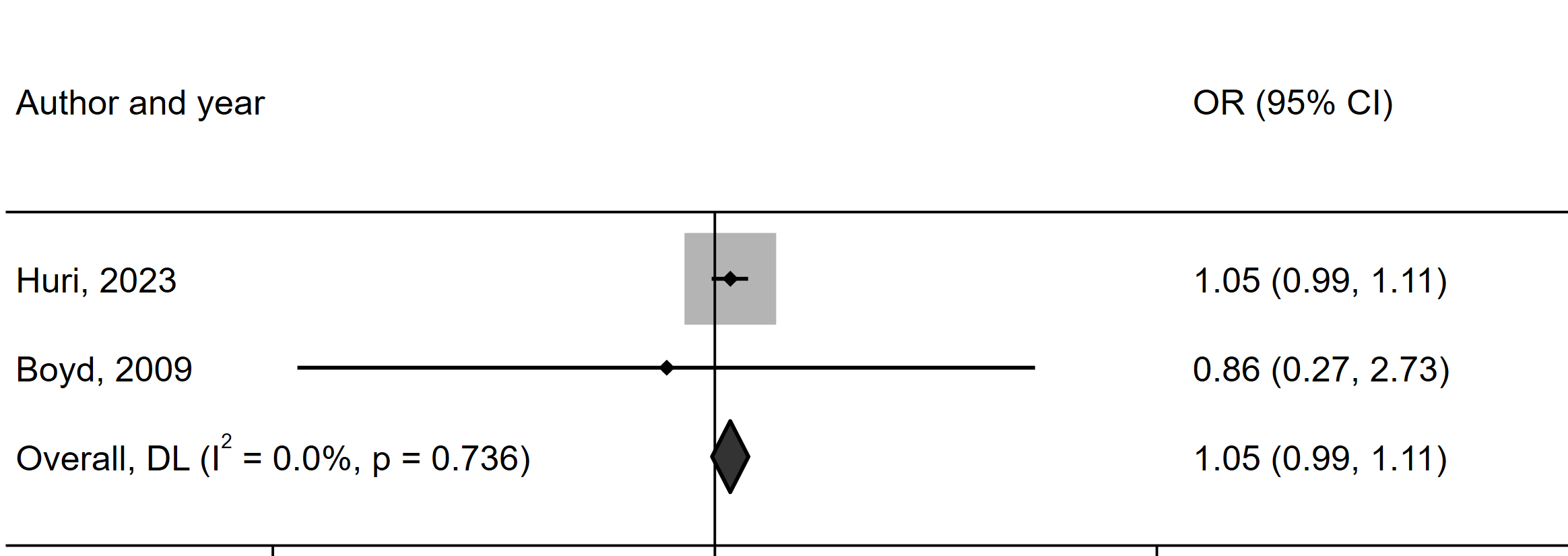

Supplement: Supplementary file 1 — Appendix S1. Table S1. Table S2. Table S3. Table S4. Figure S1. Figure S2. Figure S3. Figure S4. Figure S5. Figure S6. Figure S7. Figure S8. Figure S9. [file BJO-132-18-s001.docx]
